# Development and Clinical Evaluation of a Rapid Point of Care Test for Ebola Virus Infection in Humans

**DOI:** 10.3390/v15020336

**Published:** 2023-01-25

**Authors:** Zheng Wang, Richard S. Bennett, Michele Roehler, Geraldine Guillon, Mark J. Fischl, Mary C. Donadi, Jim Makovetz, Natalie Holmes, Toral Zaveri, Eamon Toolan, Heather L. Gontz, Graham D. Yearwood, James Logue, J. Kyle Bohannon, Lisa Mistretta, Russell Byrum, Dan Ragland, Marisa St. Claire, Lisa A. Kurtz, Tiffany Miller, Michael R. Reed, Janean Young, John Lee, Lisa E. Hensley, Keith Kardos, Jody D. Berry

**Affiliations:** 1 OraSure Technologies, Inc., Bethlehem, PA 18015, USA; 2 Bristol Myers Squibb, Princeton, NJ 08540, USA; 3 Integrated Research Facility at Fort Detrick, National Institute of Allergy and Infectious Diseases, National Institutes of Health, Frederick, MD 21702, USA; 4 Biomedical Advanced Research and Development Authority (BARDA), U.S. Department of Health & Human Services, Washington, DC 20201, USA

**Keywords:** Ebola virus, diagnosis, rapid antigen test

## Abstract

The genus *Ebolavirus* contains multiple species of viruses that are highly contagious and lethal, often causing severe hemorrhagic fever. To minimize the global threat from Ebola virus disease (EVD), sustainable, field-appropriate tools are needed to quickly screen and triage symptomatic patients and conduct rapid screening of cadavers to ensure proper handling of human remains. The OraQuick^®^ Ebola Rapid Antigen Test is an in vitro diagnostic single-use immunoassay for the qualitative detection of Ebola virus antigens that detects all known species within the genus *Ebolavirus*. Here, we report the performance of the OraQuick^®^ Ebola Rapid Antigen Test and provide a comparison of its performance with other rapid diagnostic tests (RDTs) for EVD. OraQuick^®^ Ebola demonstrated clinical sensitivity of 84.0% in archived EVD patient venous whole-blood (WB) samples, 90.9% in Ebola virus-infected monkey fingerstick samples, and 97.1% in EVD patient cadaver buccal swabs, as well as clinical specificity of 98.0–100% in venous WB samples and 99.1–100% in contrived saliva samples. It is the only 510(k)-cleared Ebola rapid test, has analytical sensitivity as good as or better than all RDT comparators for EVD, and can detect the Sudan virus. Our data demonstrate that the OraQuick^®^ Ebola Rapid Antigen Test is a sensitive and specific assay that can be used for rapid detection of EBOV in humans and could support efforts for EVD-specific interventions and control over outbreaks.

## 1. Introduction

Emerging infectious diseases continue to contribute to human morbidity and mortality. In remote areas of the developing world, humans who live in close proximity to animal reservoirs are at risk of potential exposure to zoonotic pathogens and disease vectors. The genus *Ebolavirus* belongs to the viral family *Filoviridae*, which includes six (6) species: *Zaire ebolavirus* (EBOV), Sudan *ebolavirus* (SUDV), *Bundibugyo ebolavirus* (BDBV), *Tai Forest ebolavirus* (TAFV), *Reston ebolavirus* (RESTV), and *Bombali ebolavirus* (BOMV). EBOV, SUDV, BDBV, and TAFV often cause severe and/or fatal diseases in humans, whereas RESTV and BOMV are apparently not pathogenic to humans. Since the first reports of human diseases caused by SUDV and EBOV in 1976, there have been more than 30 outbreaks of EVD in the subsequent 40 years [1]. Ebola virus disease (EVD) is a serious and frequently lethal illness with an average case fatality rate of 78% [1,2]. The 2013–2016 outbreak of EBOV in western African countries was the largest outbreak ever reported, infecting over 28,000 people, and causing over 11,000 deaths [3]. The August 2018 outbreak in the Democratic Republic of the Congo (COD) was the second largest outbreak ever reported [4] and the tenth outbreak in the COD since the virus was discovered [5]. On 17 July 2019, the World Health Organization (WHO) declared the COD EVD outbreak a Public Health Emergency of International Concern. By 25 June 2020, a total of 3470 EVD cases were reported with 2287 deaths (overall case fatality ratio: 66%) [6]. Small outbreaks have continued in the COD through mid-2022 (updated information from the CDC on the history of Ebola virus outbreaks), suggesting that, even after significant progress in the development of vaccines and other countermeasures, social and geopolitical pressures may diminish their potential effectiveness, and EVD could continue to be an important global public health threat. Recently, the re-emergence of the Sudan strain has become a reminder of the need to be prepared on all levels for an appropriate response to alternative Ebola strains [7]. Indeed, the Ministry of Health of the Republic of Uganda declared an Ebola virus disease outbreak on 20th September 2022 after a patient died who was infected with the rare Sudan strain of Ebola virus [8].

Although RT-PCR is highly sensitive and specific and considered the gold standard for EVD diagnosis, it requires a specialized laboratory setting, trained personnel, stable power supply, and appropriate biosafety procedures. Large-scale use of RT-PCR testing in an outbreak situation is nonpractical when rapid diagnosis is required for clinical management, isolation, and containment. Rapid diagnostic tests (RDTs), such as lateral flow assays that detect EBOV antigens, are ideal as screening tests and suitable for field testing and low-infrastructure settings, such as the clinic [1]. During the 2013–2016 EVD outbreak, rapid screening of cadavers to support safe and dignified burial practices was critical in controlling the outbreak and preventing the spread of EVD [1]. The OraQuick^®^ Ebola Rapid Antigen Test (OraSure Technologies, Inc., Bethlehem, PA, USA) was selectively used to test directly collected cadaver oral fluid samples obtained from Sierra Leone, Guinea, and Liberia, as it was the only RDT approved to detect the EBOV in contrived saliva samples. Testing oral fluids (OFs) eliminates the need for blood collection via needle puncture and reduces the risk of exposure for sample collection personnel. Currently, the OraQuick^®^ Ebola Rapid Antigen Test is the only RDT test approved for both cadaveric OF and live whole-blood (WB) samples.

In the 2018 COD outbreak, Cepheid GeneXpert Ebola real-time reverse transcriptase polymerase chain reaction (rRT-PCR) was the primary test used for patient screening, triage, and confirmation. Public health officials desire access to rapid screening tests, which, unlike molecular nucleic acid amplification tests (NAAT), do not require electricity or reagent refrigeration and can be distributed to affected areas in large numbers to generate results within minutes [9]. A simple, rapid point-of-care test could be a useful tool to quickly identify EBOV infection, particularly in hard-to reach-communities, in the event of outbreaks. In 2015, OraSure Technologies Incorporated (OTI) developed and launched the United States Food and Drug Administration (US FDA) Emergency Use Authorization (EUA) OraQuick^®^ Ebola Rapid Antigen Test for WB testing from live patients. Subsequently, in 2016, the product gained the US FDA EUA for cadaveric OF samples and WHO Emergency Use Assessment and Listing (EUAL) for both live-patient WB and cadaveric OF samples. The intended uses include WB specimens from individuals with epidemiological risk factors showing signs and symptoms of EVD and oral fluid specimens from deceased individuals who are suspected to have died from EVD. Logistical issues surrounding working with humans infected by this BL4 pathogen during an outbreak limited OraSure’s ability to test prospective samples. Thus, much like the animal rule is used for therapeutics for high-containment pathogens, the performance of the device with Ebola virus-positive fingerstick WB was established in a nonhuman primate model, which was the first-ever animal model used in the clearance of a rapid diagnostic test (RDT) for Ebola virus detection. Collectively, our data demonstrate that the OraQuick^®^ Ebola Rapid Antigen Test is a sensitive and specific assay that can be used for rapid detection of viruses from the genus *Ebolavirus* in humans and could support efforts for EVD-specific interventions and control over outbreaks. Here, we report the performance of the OraQuick^®^ Ebola Rapid Antigen Test in multiple clinical and analytical studies and provide a comparison with some of the other Ebola RDTs that have recently received EUA from the FDA.

## 2. Materials and Methods

### 2.1. Sample Specimen Collection

Clinical specimens were collected from the USA, Guinea, Sierra Leone, and Liberia by various organizations, including the WHO, the Department of Defense (DoD), and the Centers for Disease Control and Prevention (CDC). Contrived saliva samples (as a surrogate for cadaveric oral fluid) and fingerstick WB specimens were collected from participants under the protocol COLLECT-OFFS-2, approved by the Salus Institutional Review Board (IRB). Clinical specimens were also collected and tested directly through volunteer participation in the OraSure-sponsored protocol OQ-EVD-WBFS-30, which was granted initial approval by Salus IRB on 18 December 2017. Subjects were enrolled through recruitment methods established by the investigational sites, and written informed consent was obtained from the subjects (aged seven or older) prior to participation in the trial. The informed consent form contained the required elements according to 21 CFR 50 and 45 CFR 46 and was approved by an IRB. 

Details of the clinical samples for each sensitivity and specificity test are provided in the Methods section of the Supplementary Materials. The WHO recombinant VP40 reference material was evaluated to determine the sensitivity of the OraQuick^®^ Ebola Rapid Antigen Test [10]. This material (1st Reference Reagent 2016, lyophilized Ebola VP40 Antigen; Item #2016.2302) was obtained from the National Institute for Biological Standards and Control.

### 2.2. Device Testing

Device testing was performed as per the OraQuick^®^ Ebola Rapid Antigen Test Product Insert [11]. A total of 20 µL of WB sample was pipetted via calibrated or disposable pipette into the sample port on the device. Contrived saliva samples were collected either directly with the OraQuick^®^ Ebola Rapid Antigen Test or with a transport media collector [Σ-Virocult^®^ MW951S (Sigma-Aldrich, St. Louis, MO, USA) or BD Universal Viral Transport for Viruses, 220528 (Becton Dickinson, Franklin Lakes, NJ, USA)]. The device was read at 30 (−2) minutes after it was placed in the developer solution. 

### 2.3. Clinical Specificity and Sensitivity

Procurement of clinical samples and test protocols are discussed in the Supplementary Materials under the Clinical Specificity and Clinical Sensitivity section. 

### 2.4. Nonhuman Primate Testing and Evaluation 

Seronegative rhesus monkeys (referred to as nonhuman primates (NHPs) in this report) were inoculated intramuscularly with 1000 plaque-forming units (PFU) of Makona strain EBOV. Blood specimens were collected from all animals twice prior to inoculation (baseline), on days 3, 5, and 7 post-exposure, and when animals reached pre-determined clinical endpoints. Animals were anesthetized for all procedures. Venous blood was collected in BD vacutainer plastic blood collection tubes containing K_3_-ethylenediaminetetraacetic acid (EDTA) (if not specified otherwise, WB was collected in K_2_EDTA vacutainers throughout this report). Samples were mixed by gentle inversion, checked for clots with wooden applicator sticks, and any excessive clotting was noted. Peripheral blood was collected via fingerstick blood collection. Briefly, NHPs were sedated and placed in a supine position on a downdraft procedure table. The animal’s body temperature was maintained using a Bair Hugger system (3M). To improve peripheral circulation, the animal’s hand was placed in a warm water bath for 2 min and dried, and two fingers were selected and disinfected using 70% isopropyl alcohol wipes. A 4 mm Goldenrod lancet was used to puncture the tip of both the index and middle fingers, just off the center of the pad. The first drop of blood was wiped off with sterile gauze, and the next drop was collected with a disposable micropipette provided in the OraQuick^®^ Ebola Rapid Antigen Test kit. The micropipette was held horizontally, and the blood flowed automatically into the pipette to the volume indicator line (approx. 20 µL). Blood was collected from both fingers to reach the required volume. The fingerstick blood in the filled pipette was immediately deposited into the sample port on the OraQuick^®^ test device.

The blood samples were tested with the OraQuick^®^ test devices and/or the Ebola Zaire (EZ1) rRT-PCR (TaqMan^®^) (ThermoFisher, Freemont, CA, USA) assay [12] at multiple baseline draws and during the disease course. EZ1 processing was performed as described previously [13]. 

### 2.5. Analytical Studies

Probit analysis was used to determine the limit of detection (LoD) as per Clinical and Laboratory Standards Institute (CLSI) Guidelines EP17-A2. The LoD concentration selected by the probit analysis was confirmed by testing a blinded panel of 20 LoD samples, 20 negative samples, and 20 moderately reactive samples. Inclusivity was evaluated for known strains of the EBOV, which were provided by both the CDC and DoD as inactivated viruses. Testing of inactivated viruses provided by the CDC was completed at the CDC in Atlanta, GA. Further, analytical specificity was evaluated by testing viral, bacterial, and parasitic pathogens. Testing was performed with mostly live organisms unless otherwise indicated, and they were cultured as necessary. Each of the pathogens was spiked into venous WB or OF at the concentrations required. Study testing was completed at Microbac Laboratories, MicroBioTest Division, in Sterling, VA. Potentially interfering substances were tested in WB or OF spiked with recombinant VP40 antigen (rVP40 Ag) to assess their potential effect on the assay performance as per CLSI guidelines EP7-A2. rVP40 Ag (comprising the Zaire strain VP40 gene) is a recombinant protein expressed and purified from a bacterial expression system. A panel of recombinant Ebola VP40 from different variants has been designed and expressed to enable species variation testing with our devices. Reproducibility was assessed for WB, directly collected OF (saliva), and OF in viral transport media (VTM). Three sample types (negative, low positive, and moderate positive) were tested for each of the three device lots; tests were carried out twice daily over five days by three operators per site at each of three sites (OTI, Bethlehem, PA; Biological Specialty Corporation, Reading, PA; and Lehigh Valley Hospital, Allentown, PA, USA). 

### 2.6. Statistical Analysis

All analyses were performed using Statistical Analysis Software (SAS^®^) version 9.2 or later. The percentages for specificity/sensitivity were calculated as follows: % specificity = 100% × TN/(FP + TN) and % sensitivity = 100% × TP/(TP + FN), where TP is true positive, TN is true negative, FP is false positive, and FN is false negative. The confidence interval (CI) was calculated using the Clopper–Pearson (exact) method. Sensitivity and specificity are sometimes referred to as positive percentage agreement and negative percentage agreement, respectively. Probit analyses utilized the cumulative distribution frequency to determine the LoD, which was defined as the concentration calculated to yield reactive results 95% of the time. The numbers of LoD results included in the analysis varied depending upon the number of sample concentrations selected and prepared for testing. All analyses were performed using Microsoft Minitab^®^ Statistical Software version 17.1.0.

## 3. Results

### 3.1. Clinical Specificity Performance

Multiple clinical specificity studies were completed using negative human samples obtained as venous WB, fingerstick WB, directly collected contrived saliva samples, or contrived saliva samples collected in VTM from either the USA or western Africa. Contrived positive samples were added during testing to blind the operators. Studies were conducted at sites sponsored by the CDC, OTI (Lenexa, KS; Berlin, NJ; Orlando, FL; Rochester, NY; Allentown, PA (fingerstick only)), and WHO (Table 1). Details for each study are discussed in the Supplementary Materials. For WB samples (venous and fingerstick combined), the negative percent agreement ranged from 98.0% to 100%. In contrived saliva samples (direct collection and VTM combined), the negative percent agreement (specificity) ranged from 99.1% to 100%.

### 3.2. Clinical Sensitivity Performance

Multiple clinical sensitivity (percent positive agreement) studies were completed with either retrospective or contrived samples obtained as venous WB, fingerstick WB, directly collected contrived saliva samples, or contrived saliva samples collected in VTM from either the USA or western Africa. (Table 2) Details for each study are further discussed in the Supplementary Materials. Additional sensitivity and specificity data were obtained using the NHP animal model after discussion with the FDA. 

Details of the Sierra Leone study, the WHO reference panel study, and the nonhuman primate study are discussed below.

### 3.3. Sierra Leone Study: Comparison of Ebola Rapid Antigen Test and the VP40 rRT-PCR Assay

A total of 75 retrospective, remnant WB samples collected from patients in western Africa (Sierra Leone) during the 2013–2016 EVD outbreak were tested using the OraQuick^®^ Ebola Rapid Antigen Test. These samples were tested in western Africa using a US FDA EUA Ebola Virus VP40 rRT-PCR Assay [14] on the Bio-Rad CFX96 Touch Real-time PCR Detection System. The WB samples were then stored frozen until testing with the OraQuick^®^ Ebola Rapid Antigen Test. Testing was performed in a randomized, blinded manner. The performance of the OraQuick^®^ Ebola Rapid Antigen Test in comparison to the results generated by the EUA CDC Ebola Virus VP40 rRT-PCR Assay [15] (the Comparator) was calculated (Table 3). Samples were selected to ensure a wide range of cycle-threshold (CT) values were covered. At CT values < 24, the test was 100% sensitive and the sensitivity decreased at higher CT value ranges (see Table S1 in the Supplementary Materials). Overall, an inverse correlation was observed between test positivity and CT values, and these results suggest that the antigen test could identify individuals with transmissible infection (from samples with high viral load).

### 3.4. Detection of EBOV in NHP Samples with Rapid Antigen Test in Comparison to rRT-PCR Assay 

Due to challenges in obtaining Ebola patient fingerstick WB samples, an NHP model was used in the development of the OraQuick^®^ Ebola Rapid Antigen Test. Even though animal models have been employed in many previous studies [16], this was the first time that an NHP model had been used in validating an IVD diagnostic test. This was similar to the two-animal rule usually utilized for therapeutics. Detection of Makona strain EBOV with the OraQuick^®^ Ebola Rapid Antigen Test and EZ1 rRT-PCR assay was undertaken at multiple baseline draws and during disease course (Table 4). All baseline samples were negative, as animals had not yet been exposed to EBOV. The sensitivity of the OraQuick^®^ Ebola Rapid Antigen Test was analyzed based on a comparison with the EZ1 rRT-PCR assay for the detection of EBOV from venous blood draws and blood collected via fingerstick from infected NHPs. As compared to the EZ1 RT-PCR assay (DoD) [12], the OraQuick^®^ Ebola Rapid Antigen Test demonstrated a sensitivity of 81.8% (9 of 11 samples) and 90.9% (10 of 11 samples) and a specificity (negative percent agreement) of 100% (15 of 15 samples) and 93.3% (14 of 15 samples) for venous and fingerstick WB, respectively, in this animal model testing. The positive predictive values were 100% (9 of 9 samples) and 90.9% (10 of 11 samples), and the negative predictive values were 88.2% (15 of 17 samples) and 100% (14 of 14 samples) for venous and fingerstick whole blood, respectively. 

### 3.5. Ebola Virus VP40 Antigen Test Performance using WHO International Reference Panel Samples

The 1st Reference Reagent 2016, lyophilized Ebola VP40 Antigen (Item #2016.2302) was obtained from the National Institute for Biological Standards and Control (NIBSC). A total of nine samples were prepared by reconstituting the reference material as per the manufacturer’s instructions. Testing was repeated daily for three days using the same lot of OraQuick^®^ Ebola Rapid Antigen Tests. The results of the OraQuick^®^ Ebola Rapid Antigen Tests were in 100% agreement with all nine de-identified samples in the WHO proficiency panel (not shown).

### 3.6. Analytical Study Results

The LoD for the OraQuick^®^ Ebola Rapid Antigen Test was determined using rAg VP40, Mayinga EBOV strain ZZXDK901/812094/VSP (provided by the CDC); and gamma-irradiated EBOV (Mayinga strain; BEI, NR-31807). The LoD of the OraQuick^®^ Ebola Rapid Antigen Test was determined in different sample matrices (WB, directly collected OF, and OF in two types of VTM) for rAg VP40 and inactivated EBOV.

Several collection transport media and collection containers were compared for the effects on LoD. When using rAg VP40, the LoDs were 0.106 µg/mL, 6.51 µg/mL, and 31.98 µg/mL in directly collected OF, OF in the BD collection system, and OF in the Ʃ-Virocult^®^ collection system, respectively. The LoD for the rAg VP40 analyte in WB was 0.053 µg/mL. After correction for the sample volume per test in each matrix, the OraQuick^®^ device LoD in OF was 7 times more sensitive than the LoD in WB, 61 times more sensitive than the OF in the BD collection system, and 422 times more sensitive than the OF in the Σ-Virocult^®^ collection system. The LoD in WB for the inactivated Ebola virus preparation was determined to be 1.64 × 10^6^ TCID_50_/mL. 

The OraQuick^®^ Ebola Rapid Antigen Test was next tested for the ability to recognize antigenically distinct strains of Ebola. The inclusivity of the OraQuick^®^ Ebola Rapid Antigen Test was determined only for known species of the genus *Ebolavirus* responsible for human infection in the past. The test was reactive for EBOV, SUDV, and BDBV (major outbreaks of EVD in humans have been attributed to EBOV, SUDV, and BDBV) and non-reactive for TAFV and RESTV (Table 5). 

An additional panel of 15 inactivated biosafety level (BSL) 3 and 4 organisms (including known species of the genus *Ebolavirus*) was tested for inclusivity and cross-reactivity in WB. The results confirmed the inclusivity results (see data in Supplementary Materials, Table S6). Further, an additional 57 bacteria, parasites, viruses from the genus *Ebolavirus*, and other viruses were tested for in either WB or OF, and no cross-reactivity was observed (Tables S6 and S7). The impact of interfering substances present in WB samples on the performance of the OraQuick^®^ Ebola Rapid Antigen Test was evaluated. No interference was observed with bilirubin, hemoglobin, protein, human anti-mouse antibody, sulfamethoxazole, efavirenz, elvitegravir, chloroquine, atovaquone/progaunil, ibuprofen, acetaminophen, rifampin, biotin, erythromycin, tetracycline, aspirin, salicylic acid, amoxicillin, antinuclear antibodies (ANAs), and cholesterol at the select concentrations tested (Table S8). Neat saliva samples (as a surrogate for contrived saliva samples) were then used to evaluate the effects of interfering substances, such as mucin, leukocytes, and directly collected OF samples, on toothbrushing using the OraQuick^®^ Ebola Rapid Antigen Test. No interference was observed for these substances at the concentrations tested when spiked into OF samples or after the use of toothpaste (Table S9). The results of the OraQuick^®^ Ebola Rapid Antigen Test were highly reproducible across device lots, test sites, test operators, and test days for the WB and OF collected directly (as saliva) and in VTM. The test was highly reproducible over three sites, five days (two runs/day), multiple operators, and three sample types (Table S10). 

As of May 2018, six EBOV RDTs were listed for the detection of EBOV by stringent regulatory authorities (WHO EUAL, FDA EUA, or European Commission CE marking) [3]. Table S11 summarizes the performances of the four RDTs, including the OraQuick^®^ Ebola Rapid Antigen Test, listed for WHO EUAL and/or FDA EUA and/or approved for FDA 510(k) [1,17]. Overall, the data show that the performance of the OraQuick^®^ Ebola Rapid Antigen Test is equivalent to the other commercial tests. The OraQuick^®^ Antigen Ebola Rapid Antigen Test and DPP Ebola Antigen System (Chembio Diagnostic Systems, Inc.) are the only two rapid tests with FDA EUA authorization; however, the EUA approvals were phased out with the OraQuick^®^ 510(k) clearance. The FDA EUA was withdrawn for the ReEBOV Rapid Antigen test in May 2018 at the request of the manufacturer Corgenix. In addition, SD Biosensor’s SD Q Line Ebola Zaire Ag test, a rapid antigen test for whole blood, plasma, and serum from live patients, has WHO EUAL approval. 

## 4. Discussion 

The OraQuick^®^ Ebola Rapid Antigen Test is a reproducible, sensitive, and specific assay for the detection of EBOV in humans. This test was used in 2015 and 2018 in the western Africa and the COD EVD outbreaks, respectively. A target product profile (TPP) for EBOV diagnostics was developed by the Foundation for Innovative New Diagnostics (FIND), Doctors without Borders, the WHO, and partners in 2014 [18]. The “acceptable” TPP criteria include sensitivity >95% and specificity >99%, and the “desired” TPP criteria include sensitivity >98% and specificity >99%. There are no point of care RDTs that meet the “desired” or “acceptable” criteria listed in the TPP summarized in [1]. Two NAATs for the genus *Ebolavirus* meet the “acceptable” TPP criteria and one meets the “desired” TPP criteria [1,9,14] for species that require expensive equipment and trained personnel [19]. Automated NAATs, such as the GeneXpert and Filmarray systems, provide rapid, sample-to-answer results with minimal operator dependence or potential for cross-contamination. However, their implementation in decentralized health-care settings will meet operational challenges [20]. A point-of-care (POC) RDT, albeit less sensitive and specific than RT-PCR, would still stand to confer substantial benefits for case management and infection control efforts (especially if used in combination with RT-PCR testing) and should improve the utilization of limited clinical and public health resources [20].

The roles of NAAT and RDT in diagnosis are viewed as complimentary. Rapid point-of-care tests have great utility when new cases of suspected EBOV occur in remote locations and sample transport to a PCR testing lab would delay an immediate answer on whether the outbreak has spread. Employment of a POC RDT would be timelier under these circumstances. Presumptive positive samples can be confirmed later by PCR; meanwhile, those positive results should trigger immediate patient isolation and contact tracing to prevent disease spreading at the location. The outbreak in the eastern COD emerged in a complex political and security environment [21]. Factors affecting the outbreak included population movement in highly densely populated areas; inadequate infection and prevention control practices in many health facilities; and continued reluctance in the community [22]. The development of diagnostic tests that can be easily and rapidly deployed in emergency situations and austere environments remains a high priority for future viral outbreak containment [23], and such tests would be a crucial tool in outbreak management. In response to this need, OraSure Technologies, Inc. has developed the OraQuick^®^ Ebola Rapid Antigen Test, which has become the first FDA 510(k) RDT for the detection of viruses from the genus *Ebolavirus*. It is the first IVD test that has used animal data as part of validation for diagnostic use. Our data showed that the OraQuick^®^ Ebola Rapid Antigen Test has clinical sensitivity of 84.0% in archived EVD patient venous WB samples and 97.1% in EVD patient cadaver buccal swabs, and it has clinical specificity of 98.0–100% in venous WB samples and 99.1–100% in contrived saliva samples, suggesting the diagnostic utility of this rapid test for timely detection of EBOV infections. One caveat to this study is that the logistical issues surrounding working with humans infected by this risk group 4 pathogen during an outbreak limited the ability to test prospective samples. Other RDT tests, such as the QuickNavi-Ebola, show promise and have overlapping CT sensitivity; however, this test targets the NP protein rather than the VP40 [24]. A more direct comparison may be the CORIS EBOLA Ag K-Set, which also targets the VP40 and showed similar performance in a field laboratory [25].

The utility of the Ebola rapid detection assay makes the platform well-suited for detecting acute infections in a self-test format that can be deployed anywhere and can respect quarantine and social distancing. The OraQuick^®^ Ebola Rapid Antigen Test is fast (30 min) and portable, with no need for electricity. This test can used for both WB (venous and fingerstick) and contrived saliva samples. Moreover, it has broad detection abilities for multiple species from the genus *Ebolavirus* (*Zaire ebolavirus*, member virus: EBOV; *Sudan ebolavirus*, member virus: SUDV, and *Bundibugyo ebolavirus*, member virus: BDBV). Due to the finite limitations affecting the sustainability of polyclonal products and normal lot variation, an ideal next iteration we are working on would be to have a monoclonal antibody. The testing results suggest that the analytical sensitivity performance of the OraQuick^®^ Ebola Rapid Antigen Test is equivalent to or better than other commercial RDTs tested in this evaluation (Table S11).

EVD outbreaks remain a great public health threat, so it is important to quickly detect EBOV infection and take prompt EVD-specific interventions for isolation, treatment, and containment. The data demonstrate that the OraQuick^®^ Ebola Rapid Antigen Test is a reproducible, sensitive, and specific assay that can be used for the detection of EBOV in humans in both oral fluid and whole blood and could support efforts to control the spread of EBOV infections and outbreaks.

## Figures and Tables

**Table 1:** Specificity performance of OraQuick^®^ Ebola Rapid Antigen Test in different sample types and matrices.

Sample Type	Patient Demographic	Negative Percent Agreement % (n/N, CI)	Site Sponsor
Venous WB	Western African, HIV-2-positive	98.6% (140/142, 95.0–99.8%)	OTI
	Western African	98.0% (49/50, 89.4–99.9%)	CDC
	NHPs	100% (15/ 15, 80.0–100%)	NIH
	Febrile patients (USA)	100% (21/21, 83.9–100%)	OTI (USA)
	Non-febrile patients (USA)	100% (205/205, 98.2–100%)	
Fingerstick WB	Febrile patients (USA)	100% (21/21, 83.9–100%)	
	Non-febrile patients (USA)	99.6% (227/228, 97.6–100%)	
	NHPs	93.3% (14/ 15, 70.0–100%)	NIH
Directly collected contrived saliva samples	Sierra Leone	99.1% (109/110, 95.0–100%)	CDC
	Guinea	100% (334/334, 98.9–100%)	
	Liberia	100% (94/94, 96.1–100%)	
Contrived saliva samples in Σ-Virocult VTM	Sierra Leone	100% (193/193, 98.1–100%)	WHO
	USA	100% (63/63, 94.3–100%)	OTI
Contrived saliva samples in BD universal VTM	USA	100% (63/63, 94.3–100%)	OTI

**Table 2:** Sensitivity performance of the OraQuick^®^ Ebola Rapid Antigen Test.

Sample Type	Virus Source	Positive Percent Agreement (n/N, CI)
Venous human WB	Spiked irradiated EBOV (BEI)	100% (25/25, 86.3–100%)
	Retrospective EVD patient sample (CDC/Sierra Leone)	84.0% (21/25, 63.9–95.5%)
Fingerstick human WB	Spiked irradiated EBOV (BEI)	100% (5/5, 47.8–100%)
Venous NHP WB	EVD-infected NHP sample (NIH)	81.8% (9/11, 50.0–100%)
Fingerstick NHP WB	EBOV-infected NHP sample (NIH)	90.9% (10/11, 60.0–100%)
Directly collected contrived saliva samples	Spiked irradiated EBOV (BEI)	95.0% (19/20, 75.1–99.9%)
Contrived saliva samples in Σ-Virocult VTM	EVD-infected cadaver contrived saliva samples (WHO/Sierra Leone)	97.1% (34/35, 85.5–99.5%)

CDC: Centers for Disease Control; contrived saliva samples: cadaver oral fluid, EBOV: Ebola virus; EVD: Ebola virus disease; NHP: nonhuman primate; NIH: National Institutes of Health; VTM: viral transport medium; WB: whole blood; WHO: World Health Organization.

**Table 3:** Ebola Rapid Antigen Test agreement compared to the CDC Ebola Virus VP40 rRT-PCR Assay.

	Percent Agreement (n/N)	95% CI *
Positive Percent Agreement	84.0% (21/25) §	63.9–95.5%
Negative Percent Agreement	98.0% (49/50)	89.4–99.9%

* Calculated using Clopper–Pearson exact method; § includes specimens tested in an rRT-PCR CT range of 15–34 (refer to Table S1 containing the percent agreement for select CT ranges in the Supplementary Materials).

**Table 4:** Assay performance summary for the OraQuick^®^ assay using venous and fingerstick NHP blood compared to the EZ1 RT-PCR assay.

OraQuick^®^ Ebola Rapid Antigen Test Results by Day (Total Diagnosis/Total Tested)										
Study Day	True Positive ^a^		False Positive ^b^		True Negative ^c^		False Negative ^d^		Assay Failure ^e^	
	Venous	Finger	Venous	Finger	Venous	Finger	Venous	Finger	Venous	Finger
BL-1	0/0	0/0	0/5	0/5	5/5	5/5	0/0	0/0	0/5	0/5
BL-2	0/0	0/0	0/5	0/5	5/5	5/5	0/0	0/0	0/5	0/5
3	0/0	0/0	0/5	1/5	5/5	4/5	0/0	0/0	0/5	0/5
5	3/5	5/5	0/0	0/0	0/0	0/0	2/5	0/5	0/5	0/5
7	4/4	4/4	0/0	0/0	0/0	0/0	0/4	0/4	0/4	0/4
7 (T)	1/1	1/1	0/0	0/0	0/0	0/0	0/1	0/1	0/1	0/1
8 (T)	1/1	0/1	0/0	0/0	0/0	0/0	0/1	0/1	0/1	1/1
Total	9/11 (81.8%)	10/11 (90.9%)	0/15 (0.0%)	1/15 (6.7%)	15/15 (100%)	14/15 (93.3%)	2/11 (18.2%)	0/11 (0.0%)	0/26 (0.0%)	1/26 (3.8%)

^a^ A true positive was a sample that tested positive with the OraQuick^®^ Ebola Rapid Antigen Test and was known to be positive via RT-PCR; ^b^ false-positive samples that tested positive with the OraQuick^®^ Ebola Rapid Antigen Test but were known to be negative (unexposed or RT-PCR-negative); ^c^ true-negative samples that tested negative with the OraQuick^®^ Ebola Rapid Antigen Test and were known to be negative (unexposed or RT-PCR-negative); ^d^ false-negative samples that tested negative with the OraQuick^®^ Ebola Rapid Antigen Test but were known to be positive via RT-PCR; ^e^ assay failures were samples that did not reach the control line on the OraQuick^®^ Ebola Rapid Antigen Test or for which sample collection failed; Makona strain EBOV was used for comparison of the two assays; BL: baseline; T: terminal day.

**Table 5:** Detection of inactive viruses from the genus *Ebolavirus* with Ebola Rapid Antigen Test in whole blood.

Virus Name (Virus Origin)	Concentration Tested	Reactivity (Positive (P)/Negative (N))
Mayinga EBOV strain ZZXDK901/812094/VSP	1.5 × 10^6^ TCID_50_/mL	P ^B^
TAFV (COTE D’IVOIRE 11/27/94)	Unknown (1:10 dilution) Unknown (1:1 dilution)	N N
TAFV	1.36 × 10^7^ VP/mL 7.5 × 10^7^ VP/mL	N N
RESTV (119876 Pennsylvania)	3.16 × 10^6^ PFU/mL 3.16 × 10^7^ PFU/mL	N N
RESTV (Ebola virus/*M.fascicularis*/USA/1989/Reston-H28-R4387L)	5.83 × 10^5^ PFU/mL	N
SUDV, Boneface strain	10^5^ PFU/mL 5 × 10^5^ PFU/mL	N P ^A^
SUDV ^E^ (200011676 GULU)	5.6 × 10^4^ PFU/mL 2.8 × 10^5^ PFU/mL	P ^A^ P
SUDV Gulu strain ^E^ (2000011676)	3.25 × 10^5^ PFU/mL 5.95 × 10^5^ PFU/mL 1.19 × 10^6^ PFU/mL 1.79 × 10^6^ PFU/mL	N N P P ^C^
BDBV ^F^ Virus (200706291 UGANDA prototype)	3.98 × 10^4^ PFU/mL 1.99 × 10^5^ PFU/mL	P P
BDBV ^F^ (Uganda)	3.73 × 10^4^ PFU/mL 6.83 × 10^4^ PFU/mL 1.37 × 10^5^ PFU/mL 2.05 × 10^5^ PFU/mL	N N ^D^ P P

^A^ Two of three replicates tested positive, and acceptance criteria were met; ^B^ testing completed in LoD study; ^C^ a fourth replicate was added to confirm the original non-reactive results for one replicate—three out of four replicates were reactive; ^D^ one of three replicates was reactive, and criteria were not met; ^E,F^ the two tested materials from the same strain were obtained from different sources. Differences in preparation methods can lead to differences in the numbers of plaque-forming units, even if the starting material is the same.

## Data Availability

The data supporting the results in this study are provided in the Supplementary Materials, Tables S1–S11.

## Supplementary Materials

The following supporting information can be downloaded at: https://www.mdpi.com/article/10.3390/v15020336/s1, Table S1: Percent positive agreement of the OraQuick^®^ Ebola Rapid Antigen Test with real-time RT-PCR Assay for select CT ranges; Table S2: Percent agreement of the OraQuick^®^ Ebola Rapid Antigen Test to detect Ebola virus-spiked blood; Table S3: Concordance of OraQuick^®^ Ebola Rapid Antigen Test with Xpert Ebola Test; Table S4: Comparison of testing of cadaver oral fluid by OraQuick^®^ Ebola Rapid Antigen Test and RT-qPCR Assay; Table S5: Summary of the OraQuick^®^ Ebola Rapid Antigen Test Concordance; Table S6: OraQuick^®^ Ebola Rapid Antigen Test results with bacteria, parasites, and viruses spiked into EBOV-negative WB samples; Table S7: OraQuick^®^ Ebola Rapid Antigen Test results of bacteria and viruses spiked in EBOV-negative oral fluid samples; Table S8: Testing for interference of substances when spiked into WB samples; Table S9: Potential for interference of substances when spiked into EBOV-positive and negative oral fluid samples; Table S10: Summary of reproducibility result concordance per sample reactivity type; Table S11: Comparison of four listed Ebola RDT tests.
