# Supplementary material for: Development and Clinical Evaluation of a Rapid Point of Care Test for Ebola Virus Infection in Humans
Source: Viruses. 2023 Jan 25;15(2):336. doi: 10.3390/v15020336 (PMC9961446; doi:10.3390/v15020336)
Supplement: Supplementary file 1 [file viruses-15-00336-s001.zip › viruses-2176414-supplementary.pdf]

Supplementary Materials

Methods

Samples for testing clinical and analytical specificity and sensitivity

Western Africa retrospective HIV-2-positive plasma samples

A total of 142 samples were collected from the Ivory Coast during 2009. Since EBOV infection was not evident within the population during this period, these samples were evaluated with the OraQuick® Ebola Rapid Antigen Test Devices for specificity assessment. Testing of the OraQuick® Ebola Rapid Antigen Test was performed by OTI at its facility in Bethlehem, PA.

Whole blood and fingerstick blood samples from US sites.

A total of 493 individuals were enrolled across four sites in the United States. Of these, 38 samples were used to contrive positive samples for blinding purposes, whereas 1 individual withdrew consent, and 4 samples were excluded from the analysis due to deviations. The samples from the remaining 450 individuals were included in the analysis, of which 226 were venous WB samples and 224 were fingerstick blood samples. A subset of the individuals (21 venous WB and 21 fingerstick WB) enrolled were febrile (>38°C). Samples were tested with the OraQuick® Ebola Rapid Antigen Test at four clinical study sites: Legacy Pediatrics, Rochester, NY; Johnson County Clinical Trials, Lenexa, KS; Hassman Research Institute, Berlin, NJ; and Bioclinica Research, Orlando, FL.

Testing in whole blood samples spiked with irradiated Ebola virus

A total of 25 blood samples from febrile individuals ($\geq$38℃) were collected and spiked with EBOV (Mayinga, Gamma-Irradiated, NR-31807, BEI) variant to make positive contrived samples. A total of 12 of the samples were spiked at 1.5 x LoD. The remaining 13 samples were spiked at 5 x LoD. These spiked samples were used to evaluate the performance the OraQuick® Ebola Rapid Antigen Test. For blinding purposes, negative samples were also tested. All samples were tested in single replicates (n = 1) and read at 30 (-2) minutes.

WHO EUAL comparison of rapid diagnostic tests

A study conducted by WHO for Emergency Use Assessment and Listing for Ebola Virus Disease In Vitro Diagnostics (EVD IVD) evaluated products from three commercially available antigen detection assays: SD Ebola Zaire Ag (SD Biosensor), DPP Ebola Antigen test (Chembio), and OraQuick® Ebola Rapid Test (Orasure). The three assays were tested in parallel and compared to the results obtained using the Cepheid Xpert® Ebola Test at the Nigerian/European Union Mobil Laboratory Consortium (EMLab) reference laboratory. This laboratory was involved in the management of EVD testing in Kambia, Sierra Leone between October and November 2015. Oral fluid swab specimens were retrospectively and prospectively collected from cadavers in Western Africa in 2015.

Archived contrived saliva samples specimens that were PCR-positive for EBOV RNA were provided by Public Health England and specimens negative for EBOV RNA by PCR were provided by the Nigerian and Hastings EMLab labs. Specimens were collected in Σ-Virocult® System (MW951S) VTM (United Tissue Network). Of the 55 RealStar Filovirus Screen RT-PCR-positive specimens tested on Xpert® Ebola Assay (Cepheid), a total of 51 specimens were positive, 3 were negative, and 1 specimen was invalid. A total of 16 specimens were of low volume and

were diluted 1 to 4 with deionized water prior to PCR and RDT testing. A final total of 35 positive specimens were included in the RDT evaluation. A total of 193 RealStar Filovirus Screen RT-PCR-negative specimens were tested on the Xpert Ebola Assay (Cepheid for the evaluation of the RDTs.

Cadaveric oral fluid samples in viral transport medium

A matched set of 63 contrived saliva samples samples were collected in both Becton Dickinson (BD) Universal Viral Transport for Viruses and Σ-Virocult® virus specimen transport System. All samples were collected within 96 hours of intake of the cadaver, shipped frozen, and stored at -80°C. A total of 10 contrived samples with Ebola rVP40 Ag were included in the testing.

CDC Study of directly collected cadaveric oral fluid samples

CDC, in collaboration with the Ministry of Health (MOH), conducted a field evaluation of RDTs to detect EBOV in contrived saliva samples. A total of 111 samples from Sierra Leone were tested with the OraQuick® Ebola Rapid Antigen Test according to the instructions for use with contrived saliva samples using the direct sampling method. In Guinea and Liberia, 334 and 97 contrived saliva samples swab samples collected from June 2015 to August 2015 were tested and with the OraQuick® Ebola Rapid Antigen Test in parallel with CDC or the DoD EUA PCR test.

Saliva-spiked irradiated Ebola virus

A total of 25 saliva samples from live individuals were collected as oral fluid surrogates for cadavers and spiked with EBOV (Mayinga variant, Gamma-Irradiated, NR-31807, BEI) to make positive contrived samples. A total of 10 of the samples were spiked at 1.5 x LoD, whereas 10

samples were spiked at 5 x LoD. The remaining 5 samples remained unspiked to provide EBOV-negative samples to blind the reads.

RT-PCR testing

Nucleic acid extracts of each dilution of live EBOV and inactivated EBOV were tested at the CDC in a BSL-2 laboratory by RT-PCR as described in the Ebola Virus VP40 Real-time RT-PCR Assay EUA Package Insert. For the NHP study, nucleic acid extracts of inactivated EBOV, Makona variant collected from each infected NHP were tested by RT-PCR as described in the Ebola Zaire (EZ1) rRT-PCR (TaqMan®) Assay Package Insert.

Results

Clinical Specificity and Sensitivity Results

Table 1, Table 2 and Table 3 are discussed in detail in this section of the Supporting Information

Western Africa HIV-2 positive plasma samples

A total of 142 HIV-2-positive plasma samples were tested on the OraQuick® Ebola Rapid Antigen Test. A total of 140 out of 142 samples were non-reactive, and 2 samples had "N" at the 30- minute read. N is defined as a Test Line that is above negative but still below the LoD (1+) of the device. These two samples were repeated in replicates of five (n=5) and yielded similar results. Specificity was calculated to be 98.6% (140/142; 95.0% CI: 95.0% – 99.8%).

Whole blood and fingerstick whole blood samples from US sites

In the venous samples (n=226), specificity was 100% in the febrile and non-febrile groups). In fingerstick samples (n=224), specificity was 100% in the febrile group and 99.6% in the non-

febrile group. The nonfebrile group samples had 1 false-positive sample, which the reference central laboratory confirmed as negative.

Retrospective Ebola virus disease patient samples (Sierra Leone)

A total of seventy-five (75) remnant WB samples collected from patients in Western Africa (Sierra Leone) during the 2013–2016 EVD outbreak were tested using the OraQuick® Ebola Rapid Antigen Test. A breakdown of percent agreement of the two tests by CT ranges are summarized in Table S1.

Table S1: Percent positive agreement of the OraQuick® Ebola Rapid Antigen Test with real-time RT-PCR Assay for select CT ranges.

RT-PCR CT Ranges	Percent Agreement	95% CI
15 – 24	100% (16/16)	86.77% – 100%
15 – 29	90.5% (19/21)	69.6% – 98.8%
15 – 34	84.0% (21/25)	63.9% – 95.5%

Testing in whole blood spiked with irradiated Ebola virus

WB venous and fingerstick samples, spiked with gamma-irradiated Ebola virus (BEI, NR-31807), were used to evaluate the performance the OraQuick® Ebola Rapid Antigen Test. Percent Positive Agreement for both the venous WB and the FSWB were 100% (Table S2:). The negative samples yielded expected results with 100% concordance (data not shown).

Table S2: Percent agreement of the Oraquick® Ebola Rapid Antigen Test to detect Ebola virus-spiked blood.

Sample Type	Percent Agreement (n/N)	95% CI*
Venous WB	100% (25/25)	86.3% - 100%
Fingerstick WB	100% (5/5)	47.8% - 100%

n = number in agreement, N = sample size

* Confidence Interval (CI) calculated using Clopper-Pearson (exact) method.

WHO study of cadaver oral fluid retrospective specimens

A total of 35 positive and 193 negative retrospective cadaveric specimens (collected in VTM) were tested in a study conducted by the WHO. Using the Xpert Ebola Test (Cepheid) as the comparator assay, the performance of the OraQuick® Ebola Rapid Antigen Test is shown in the following Table S3.

Table S3: Concordance of OraQuick® Ebola Rapid Antigen Test with Xpert Ebola Test.

	**Xpert Ebola Test (Cepheid)**	
	**Number of positive samples Positive percent agreement (95% CI)**	**Number of negative samples Negative percent agreement (95% CI)**
**OraQuick® Ebola Rapid Antigen Test**	34/35<br>97.1%<br>(85.5% – 99.5%)	193/193<br>100%<br>(98.1% – 100%)

CDC study of cadaver oral fluid– Sierra Leone, Guinea, and Liberia

CDC, in collaboration with the Ministry of Health (MOH), conducted a field evaluation of OraQuick® Ebola Rapid Antigen Test for testing cadaver fluid. A total of 111 samples tested by OraQuick® RDTs were all negative by RT-qPCR (the 50 specimens collected in Kenema were tested using the Trombley RT-qPCR assay on the Cepheid Smartcycler II). Three samples that initially produced invalid results were re-tested according to the instructions for use; two of these samples were re-tested as negative. One of the invalid results could not be resolved by re-testing and was therefore excluded from the performance calculation. The following table includes a summary of the field study data. This study provided an NPA for the OraQuick® Ebola Rapid Antigen Test of 109/110 = 99.1% with a 95% Confidence Interval of 95.0 – 100%.

Table S4: Comparison of testing of cadaver oral fluid by Oraquick® Ebola Rapid Antigen Test and RT-qPCR assay.

Date of testing	Region	RDTs tested	Reactive	Non-reactive	Invalid	RT-qPCR
January 10, 2016	Kenema, Sierra Leone	50	0	50[A]	0	50[B]
January 6 – 13, 2016	Western Africa	61	1[C]	59[D]	1[D]	61
	**Total**	**111**	**1**	**109**	**1**	**111**

A) The control line on the OraQuick® device was not visualized, and the test was interpreted as invalid. Upon repeat testing, the OraQuick® test was non-reactive. A swab for RT-qPCR was collected, and the samples were negative by RT-qPCR. B) RT-qPCR was performed in conjunction with the OraQuick® tests for all cadavers. The 111 samples tested by OraQuick® RDTs were all negative by RT-qPCR (Trombley RT-qPCR assay on the Cepheid Smartcycler II.). C) The OraQuick® results were reactive, but RT-qPCR confirmation was negative. D) Two initial OraQuick® devices were interpreted as invalid. Upon repeat testing with OraQuick®, one sample was non-reactive and the other was invalid. The twice invalided sample was tested by RT-qPCR and was found to be negative. RDT: rapid diagnostic test; RT-qPCR: reverse transcripase quantitative polymerase chain reaction.

Ebola Positive Contrived Oral Fluid Samples

The performance of the device was evaluated using twenty (n=20) Ebola virus contrived positive saliva samples as a surrogate for direct collect oral fluid. Samples were contrived positive using gamma-irradiated genus Ebola virus (BEI, NR-31807). Five (5) EBOV negative samples were included in the blinded test reads to control for bias. Percent Positive Agreement was 95%. One contrived positive sample at the 1.5 x LoD level was recorded as a non-reactive result, which leads to a Positive Percent Agreement (PPA) of 19/20 = 95% with a 95% Confidence Interval of 75.1 to 99.87%. Results are summarized in Table S5

Table S5: Summary of OraQuick® Ebola Rapid Antigen Test concordance.

Sample Type	Percent Agreement (n/N)	95% CI*
Saliva as Surrogate for Oral Fluid Direct Collect	95.0% (19/20)	75.1% - 99.9%
n = number in agreement, N = sample size<br>* Confidence Interval (CI) calculated using Clopper-Pearson (exact) method.		

Cross reactivity

The effect of cross-reactive species on the performance of the OraQuick® Ebola Rapid Antigen Test was evaluated. No cross-reactivity was observed with any of the bacteria, parasites, or viruses not in the genus *Ebolavirus* tested in whole blood (Table S6 and S7). Potentially cross-reactive organisms were spiked into WB samples. A total of three replicates were tested with the pathogens spiked into EBOV-negative WB at the concentrations listed. Due to the unavailability of *Yersinia pestis, Borrelia recurrentis, Rickettsia prowazekii*, and *Rickettsia typhi* as initially identified in the protocol, surrogate organisms *Borrelia hermsii, Yersinia pseudotuberculosis*, and *Rickettsia australis* were utilized as replacements.

Table S6: OraQuick® Ebola Rapid Antigen Test results with bacteria, parasites, and viruses spiked into EBOV- negative WB samples.

Virus/Bacteria/Parasite	Type/Strain	Concentration tested	Reactivity (n=3)
Adenovirus	Type 5 ATCC VR-5	2.0 x 10^5 $TCID_{50}$/mL	None
Bacteroides fragilis	VPI 2553 [EN-2; NCTC 9343] ATCC 25285	1.0 x 10^8 CFU/mL	None
Borrelia hermsii$^+$	HS1 Serotype 26 ATCC BAA-2821	1:11 Dilution^	None
Chikungunya virus	ATCC VR-64	3.0 x 10^8 LD_{50}/mL	None
Crimean Congo Hemorrhagic Fever*	OMAN199809166 #811466	5.6 x10^4 $TCID_{50}$/mL	None

**Virus/Bacteria/Parasite**	**Type/Strain**	**Concentration tested**	**Reactivity (n=3)**
Cytomegalovirus	AD-169 ATCC VR-1788	2.9 x 10^5 $TCID_{50}$/mL	None
Enterococcus faecium	NCTC 7171 [DSM 20477, JCM 8727, NCDO 942] ATCC 19434	7.6 x 10^7 CFU/mL	None
Enterovirus	Enterovirus 71 ATCC VR-1432	5.1 x 10^5 $TCID_{50}$/mL	None
Epstein-Barr Virus	Lymphocryptovirus P-3 ATCC VR-602	144 cp/mL (TBD by National Institute of Standards and Technology)	None
Escherichia coli	AMC 198 ATCC 11229	4.5 x 10^7 CFU/mL	None
Hemophilus influenza	ATCC 33930	3.0 x 10^7 CFU/mL	None
Hepatitis A virus	Clinical Sample, University of Ottawa 021916B1C-1D	4.5 x 10^7 $TCID_{50}$/mL	None
Hepatitis B virus	Clinical Sample, DLS13-01459	1.2 x 10^7 IU/mL	None
Hepatitis C virus	Clinical Sample, DLS14-08008	4.7 x 10^6 IU/mL	None
Human immunodeficiency virus-1	Clinical Sample, ZeptoMetrix 022117B1D	5.1 x 10^6 $TCID_{50}$/mL	None
Influenza A virus	A/Wisconsin/10/1998	2.3 x 10^5 $TCID_{50}$/mL	None
Influenza B virus	B/Florida/04/06	4.6 x 10^5 $TCID_{50}$/mL	None
Klebsiella pneumoniae	[CIP 104216, NCIB 10341] ATCC 4352	5.8 x 10^7 CFU/mL	None
Lassa virus*	Josiah	3.16 x 10^5 PFU/mL<br>1.88 x 10^5 PFU/mL	None<br>None
	Macenta (Z-136)	1.9 x 10^7 PFU/mL	None
	Pinneo	2.00 x 10^6 PFU/mL	None
Leptospira biflexa	ATCC 23582	~ 9.1 x 10^4 CFU/mL	None
Marburg virus*			
	Lake Victoria (Musoke)	3.73 x 10^6 PFU/mL	None
	200501379 Angola	1.44 x 10^6 PFU/mL	None
	Voege	3.6 x10^6 PFU/mL	None
Measles virus	Edmonston ATCC VR-24	1.5 x 10^6 $TCID_{50}$/mL	None
Mumps virus	Jones ATCC VR-1438	7.1 x 10^5 $TCID_{50}$/mL	None
Neisseria meningitides	ATCC 13090	3.5 x 10^6 CFU/mL	None
Plasmodium falciparum	ATCC 30932	0.26% parasitemia	None
Plasmodium viva	ATCC 30151	6.8 x 10^5 cells/mL	None
Pseudomonas aeruginosa	ATCC 15442	3.4 x 10^8 CFU/mL	None

Virus/Bacteria/Parasite	Type/Strain	Concentration tested	Reactivity (n=3)
RAVN virus		Log 5.53 $TCID_{50}$/mL	None
Rickettsia africae	(protein) BEI NR-42992	11.6 mg/mL	None
Rickettsia australis[+]	JC BEI NR-10454	8.1 x 10^5 $TCID_{50}$/mL	None
Rift Valley Fever virus*	ZH-501	3 x10^6 pfu/mL<br>5.45 x 10^6 PFU/mL	None<br>None
Rotavirus	ATCC VR-899	6.4 x 10^6 $TCID_{50}$/mL	None
Respiratory syncytial virus	ATCC VR-26	9.0 x 10^5 $TCID_{50}$/mL	None
Rubella virus	M33 ATCC VR-315	1.5 x 10^5 $TCID_{50}$/mL	None
Salmonella enterica	ATCC 10708	3.8 x 10^7 CFU/mL	None
Salmonella typhi	ATCC 6539	5.4 x 10^5 CFU/mL	None
Shigella dysenteriae	ATCC 9361	4.0 x 10^6 CFU/mL	None
Streptococcus pneumonia	CIP 104225ATCC 6303	2.1 x 10^8 CFU/mL	None
Streptococcus pneumoniae	Slovakia 14-10 [29055] ATCC 700677	4.6 x 10^4 CFU/mL	None
Trypanosoma cruzi	ATCC 30013	3.1 x 10^7 CFU/mL	None
Vesicular Stomatitis virus	Indiana Lab [V-520-001-522] ATCC VR-158	7.9 x 10^6 $TCID_{50}$/mL	None
Vibrio cholera	ATCC 39050	6.7 x 10^5 CFU/mL	None
West Nile virus	B 956 [V-554-001-522] ATCC VR-1267	2.3 x 10^8 $TCID_{50}$/ml	None
Yellow fever virus*	Vaccine Strain #806588<br>Asibi	Unknown^<br>1.88 x 10^6 PFU/mL	None<br>None
Yersinia enterocolitica	ATCC 23715	7.0 x 10^6 CFU/mL	None
Yersinia pseudotuberculosis[+]	IP2666 BEI NR-4371	1:11 Dilution^	None

* = Organisms and strains marked with an asterisk were inactivated.
[+] = surrogate organisms
^= stock concentration undetermined

In addition, potentially cross-reactive organisms were tested in the contrived saliva samples. In these studies, three replicates were tested with the live pathogens spiked into EBOV-negative oral fluid (saliva as surrogate) at the concentrations listed (Table S7). None of the tested organisms produced false positive results using the OraQuick® Ebola Rapid Antigen Test.

Table S7: OraQuick® Ebola Rapid Antigen Test results of bacteria and viruses spiked in EBOV-negative oral fluid samples.

Virus/Bacteria	Type/Strain	Concentration tested	Reactivity
Actinomyces viscosus	ATCC 43146	2.9 x 10^6 CFU/mL	None
Bacteroides oralis	VPI D27B-24 [NCTC 11459] ATCC 33269	5.1 x 10^5 CFU/mL	None
Bordetella pertussis	Tohama I ATCC	6.9 x 10^6 CFU/mL	None
Candida albicans	ATCC 18804	6.1 x 10^6 CFU/mL	None
Chlamydolphia	AR-39 ATCC 53592	2.6 x 10^6 IFU/mL	None
Corynebacterium	5159 ATCC 13812	8.4 x 10^6 CFU/mL	None
Herpes simplex virus	ATCC VR-260	2.7 x 10^6 $TCID_{50}$/mL	None
Herpes simplex virus 2	ATCC VR-734	1.0 x 10^6 $TCID_{50}$/mL	None
Lactobacillus johnsonii	ATCC 33200	1.9 x 10^6 CFU/mL	None
Moraxella catarrhalis	Ne 11 [CCUG 353,LMG 11192,	2.7 x 10^7 CFU/mL	None
Mycobacterium	ATCC 25177	1.0 x 10^6 CFU/mL	None
Mycoplasma pneumonia	FH strain of Eaton Agent [NCTC 10119]	2.6 x 10^6 CFU/mL	None
Nocardia sp.	N1408 [QN360]	5.4 x 10^5 CFU/mL	None
Porphyromonas	ATCC 49417	1.7 x 10^6 CFU/mL	None
Staphylococcus aureus	ATCC 6538	3.6 x 10^7 CFU/mL	None
Staphylococcus	ATCC 12228	3.9 x 10^6 CFU/mL	None
Streptococcus mutans	ATCC 25175	2.0 x 10^6 CFU/mL	None
Streptococcus pyogenes	ATCC 19615	1.4 x 10^7 CFU/mL	None
Streptococcus salivarius	ATCC 7073	7.7 x 10^6 CFU/mL	None

Interference

Interference is a cause of clinically significant bias in the measured analyte concentration because of another component or property of the sample. The impact of interfering substances present in WB samples on the performance of the OraQuick® Ebola Rapid Antigen Test was evaluated. No interference was observed with bilirubin, hemoglobin, protein, hama, sulfamethoxazole, efavirenz, elvitegravir, chloroquine, atovaquone/progaunil, ibuprofen, acetaminophen, rifampin, biotin,

erythromycin, tetracycline, aspirin, salicylic acid, amoxicillin, and cholesterol at the concentrations tested in Table S8. Rheumatoid Factor caused false positive results at concentrations greater than 1050 IU/mL. Concentrations of Rheumatoid Factor equal to, or less than 1050 IU/mL did not cause interference. FNs observed under positive conditions (spiked with interfering substance in the presence of EBOV rVP40 Ag) were repeated at a 1:2 dilution for ribavirin, emtricitabine, ritonavir, and quinine. All interfering substances were concordant with expected results after the repeat testing. ANA caused FNs results at 1:160 titer. Repeat testing of ANA at 1:120 titer for the FNs encountered under positive conditions were concordant with expected results. The results demonstrate that ANA titers greater than 1:120 may interfere with the performance of the OraQuick® Ebola Rapid Antigen Test.

Table S8: Testing for interference of substances when spiked into WB samples.

Potential Interfering substance	Target concentration	n	EBOV-negative samples		EBOV-positive rVP40 Ag-spiked Samples	
			Reactive	Non-reactive	Reactive	Non-reactive
Bilirubin	25 mg/dL	6	0	6 (100%)	6 (100%)	0
Hemoglobin	20 g/dL	6	0	6 (100%)	6 (100%)	0
Protein	5 g/dL	6	0	6 (100%)	6 (100%)	0
Human anti-mouse antibody	2464 ng/mL	6	0	6 (100%)	6 (100%)	0
Rheumatoid Factor	2920 IU/mL	2	2 (100%)	0	2 (100%)	0
	1460 IU/mL	4	4 (100%)	0	4 (100%)	0
	1050 IU/mL	2	0	2 (100%)	2 (100%)	0
Cholesterol	13 mmol/L	12	0	12 (100%)	12 (100%)	0

Potential interfering substance	Target concentration	n	EBOV-negative samples		EBOV-positive rVP40 Ag-spiked Samples	
			Reactive	Non-Reactive	Reactive	Non-Reactive
Antinuclear Antibodies (ANA)	1:160 titer	12	0	12 (100%)	11 (91.7%)	1 (8.3%)
	1:120 titer	3			3 (100%)	0
Acetylsalicylic Acid	3.62 mmol/L	12	0	12 (100%)	12 (100%)	0
Salicylic Acid	4.34 mmol/L	12	0	12 (100%)	12 (100%)	0
Sulfamethoxazole	1.58 mmol/L	12	0	12 (100%)	12 (100%)	0
Ribavirin	11.04 µg/mL	12	0	12 (100%)	11 (91.7%)	1 (8.3%)
	5.52 µg/mL	3			3 (100%)	0
Emtricitabine	7.5 µg/mL	12	0	12 (100%)	11 (91.7%)	1 (8.3%)
	3.75 µg/mL	3			3 (100%)	0
Efavirenz	16.6 µg/mL	12	0	12 (100%)	12 (100%)	0
Ritonavir	33.6 µg/mL	12	0	12 (100%)	10 (83.3%)	2 (16.7%)
	16.8 µg/mL	6			6 (100%)	0
Elvitegravir	5.61 µg/mL	12	0	12 (100%)	12 (100%)	0
Chloroquine	17.95 µg/mL	12	0	12 (100%)	12 (100%)	0
Atovaquone/ Proguanil	89.1/2.8 µg/mL	12	0	12 (100%)	12 (100%)	0
Ibuprofen	2425 µmol/L	12	0	12 (100%)	12 (100%)	0
Acetaminophen	1324 µmol/L	12	0	12 (100%)	12 (100%)	0
Quinine	148 µmol/L	12	0	12 (100%)	11 (91.7%)	1 (8.3%)
	74 µmol/L or 24 µg/mL	3			3 (100%)	0
Rifampin	78.1 µmol/L	12	0	12 (100%)	12 (100%)	0
Amoxicillin	206 µmol/L	12	0	12 (100%)	12 (100%)	0

Tetracycline	34 µmol/L	12	0	12 (100%)	12 (100%)	0
Erythromycin	81.6 µmol/L	12	0	12 (100%)	12 (100%)	0
Biotin	3.6 µg/mL	12	0	12 (100%)	12 (100%)	0

EBOV: Ebola virus; rVP40 Ag: Ebola virus recombinant VP40 (matrix protein) antigen;

Further, the OraQuick® Ebola Rapid Antigen test was evaluated in negative oral fluid and oral fluid spiked with EBOV recombinant antigen (rVP40 Ag) at 2.0 X the LoD using the following interfering substances (toothpaste, mucin and leukocytes) to assess their potential effect on the assay performance as per CLSI guidelines EP17-A2[4]. Leukocytes. Testing was completed on three oral fluid samples each tested at n = 2 replicates for each condition. The concentration for toothpaste could not be analytically quantified, but duration of interference use was two minutes and then a 30-minute wait before direct collection using the device. At the mucin 20 mg/mL concentration, one false positive was detected in the negative oral fluid test group and one FN in the rVP40 Ag spiked oral fluid test group. No interference was noted at the 15 mg/mL test concentration (Table S9).

Table S9: Potential for interference of substances when spiked into EBOV -positive and -negative oral fluid samples.

Interfering Substances	Target Testing Concentration	Reactivity
Toothpaste	n/a	None
Mucin	20 mg/mL	Reactive
	15 mg/mL	None
Leukocytes	6.12×10^9 cells / L	None

Reproducibility

Reproducibility is measurement of precision under a set of reproducibility conditions that includes different locations, operators, measuring systems, and replicate measurements on the same or similar objects. The OraQuick® Ebola Rapid Antigen Test was highly reproducible across device lots, test sites, test operators, and test days for WB and OF in VTM (Table S10).

Table S10: Summary of reproducibility result concordance per sample reactivity type.

Sample Type	Negative		Low Positive (2x LoD)		Moderate Positive (5x LoD)	
	n/N	% (CI)	n/N	% (CI)	n/N	% (CI)
WB	805/808	99.6 (98.9, 99.9)	807/808	99.9 (99.3, 100.0)	810/810	100.0 (99.5, 100.0)
OF (saliva as surrogate)	794/809	98.1 (97.0, 99.0)	770/809	95.2 (93.5, 96.5)	804/809	99.4 (98.6, 99.8)
VTM (saliva as surrogate)	808/809	99.9 (99.3, 100.0)	807/809	99.8 (99.1, 100.0)	810/810	100.0 (99.5, 100.0)

Table S11: Comparison of four listed Ebola RDT Tests.

Test Name	OraQuick Ebola Rapid Antigen Test (12) (18) (19)	DPP Ebola Antigen System (17)	ReEBOV Antigen Rapid Test (20) (21) (22)	SD Q Line Ebola Zaire Ag (23) (24)
Manufacturer	OraSure Technologies, Inc.	Chembio Diagnostic Systems, Inc.	Corgenix, Inc.	SD Biosensor Inc.
Regulatory Status	FDA EUA, WHO EUAL, FDA 510(k) de novo	FDA EUA	WHO EUAL, FDA EUA	WHO EUAL
Target	VP40 and EBOV	VP40	VP40	GP, NP, VP40
Sample Matrices	Whole Blood (Venous and Fingerstick); Cadaveric Oral Fluid	Whole Blood (Venous and Fingerstick); EDTA plasma	Whole Blood (Venous and Fingerstick); EDTA plasma; Serum	Whole Blood; EDTA plasma; Serum
LoD (VP40 rAg spiked in Whole Blood)	53 ng/mL or 1.06 ng/test	250 ng/ mL or 12.5 ng/test	625 ng/mL or 18.8 ng/test	unknown
Reactivity to Ebola Strains	Zaire, Sudan and Bundibugyo	Zaire	Zaire, Sudan and Bundibugyo	Zaire
Sensitivity in Venous Whole Blood	84.0% (21/25), 95% CI (63.9% - 95.5%), PCR Ct range 15-34	N/A	78.3 (18/23), 95% CI (58.1% - 90.3%)	84.9% (126/149), 95%CI (78.6% - 91.2%)
Specificity in EDTA Venous Whole Blood	Febrile-100% (21/21), 95% CI (83.89%-100.0%)	98.2% (245.5/250), 95% CI (95.7%-99.3%)	90.7 (117/129), 95% CI (84.4% - 94.6%)	99.7% (289/290), 95%CI (99.1% - 100%)
	Non-febrile-100% (205/205), 95% CI (98.22%-100.0%)			
Sensitivity in Fingerstick Whole blood (Nonhuman primate)	90.9% (10/11), 95% CI (60.0% – 100%)	N/A	N/A	N/A
Specificity in Fingerstick Whole blood	Febrile-100% (21/21), 95%CI (83.89%-100.0%)	98.2% (54/55), 95% CI (90.4%-99.7%)	97.5% (39/40) (95% CI 86.8% – 99.9%)	N/A
	Non-febrile-99.6% (227/228), 95% CI (97.59%-99.99%)			
Sensitivity in Cadaveric Oral Fluid	97.1% (34/35), 95% CI (85.5%-99.5%)	N/A	N/A	N/A
Specificity in Cadaveric Oral Fluid	100.0% (193/193), 95% CI (98.1%-100%)	N/A	N/A	N/A

EUA: Emergency use authorization; EUAL: Emergency Use Assessment and Listing; FDA: Food and Drug Administration
